# P-1593. Immunogenicity and Safety of Concomitant Versus Sequential COVID-19 and Influenza Vaccination Strategies: A Systematic Review and Meta-Analysis of Randomized Controlled Trials

**DOI:** 10.1093/ofid/ofaf695.1772

**Published:** 2026-01-11

**Authors:** Anchit Chauhan, Maulinkumar Patel, Marhaba Fatima, Ishita Gupta, Muhammad Hamza, Lokesh Chauhan, Ashfaq Ahmad, Marium Zahid

**Affiliations:** Maulana Azad Medical College, New Delhi, Kurukshetra, Haryana, India; University of Texas Health Science Centre at Houston, Houston, Texas; Peoples University of Medical &Health Sciences for Women, Nawabshah, Pakistan, Nawabshah, Sindh, Pakistan; St. Bernards Medical Center, Jonesboro, Arkansas; Saidu medical college swat, Saidu sharif, North-West Frontier, Pakistan; Sarojini Naidu Medical College, Agra, Agra, Uttar Pradesh, India; Gomal Medical College, D I Khan, North-West Frontier, Pakistan; Karachi Medical and Dental College, Karachi, Sindh, Pakistan

## Abstract

**Background:**

Optimizing vaccines against co-circulating COVID-19 and influenza, especially coadministration, offers several logistical advantages. While CDC supports coadministration and uptake is rising (BNT162b2 co-administration with other respiratory vaccines: 2.7% [2021] to 34.1% [2023]), overall dual vaccine coverage of COVID and influenza vaccines remains low (19.2% US adults, 2023-24), with higher co-administration among COVID-19 vaccine recipients (48.5%) than influenza recipients (22.9%). Following WHO’s 2021 interim guidance on this, several RCTs were initiated. We conducted the first SRMA, synthesizing their results.

**Figure:**
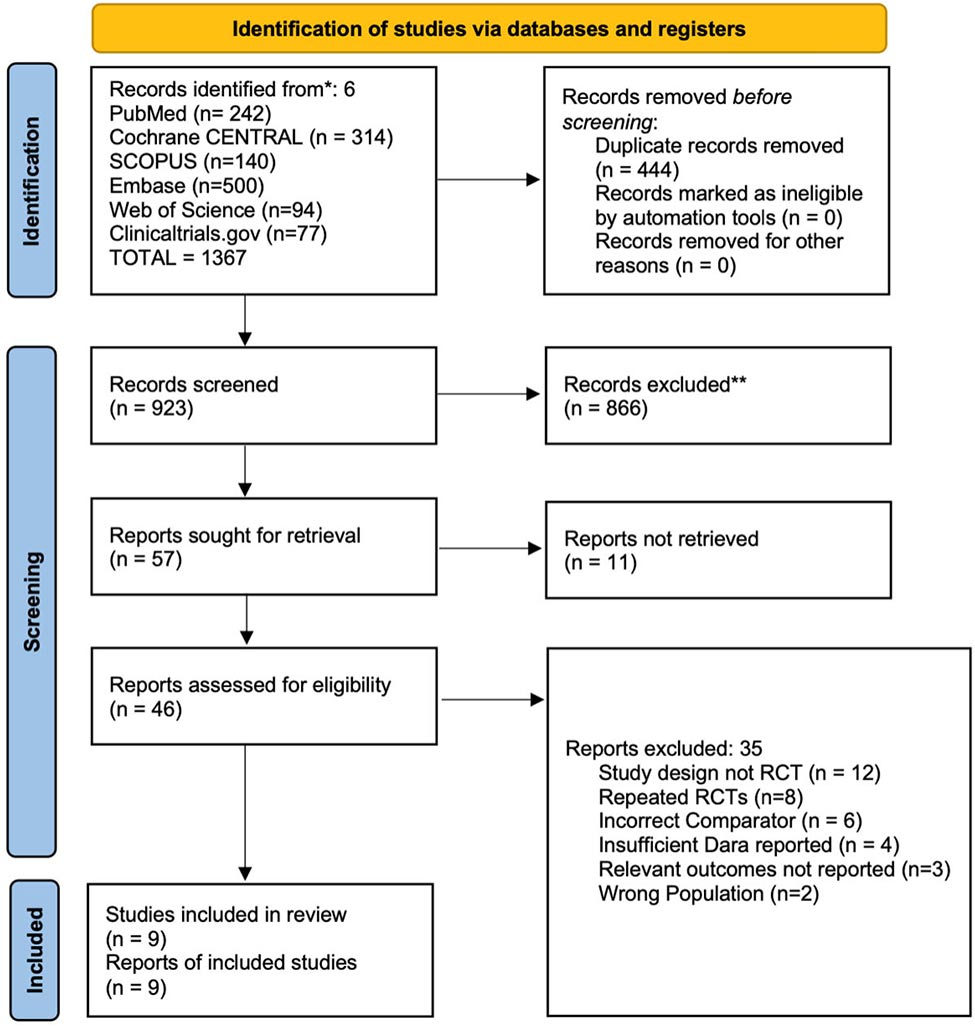
Preferred Reporting Items for Systematic reviews and Meta-Analyses Flow Chart

**Figure:**
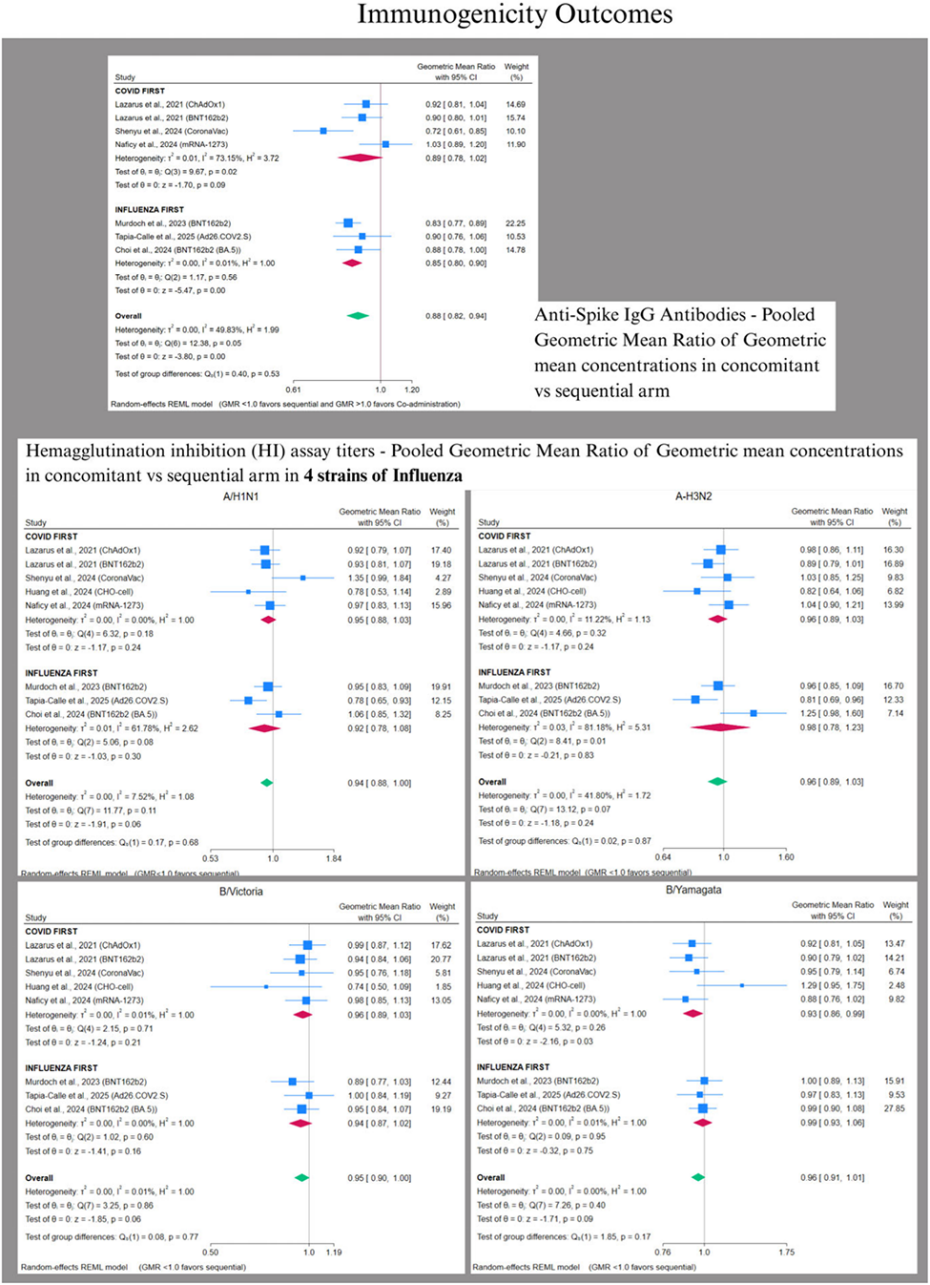

**Methods:**

We followed PRISMA guidelines (PROSPERO: CRD420251035890). PubMed, Cochrane CENTRAL, Embase, Scopus, and Web of Science were searched on 04.01.2025, and RCTs comparing concomitant (same visit) vs. sequential (separate visits, interval >0 days, influenza first or COVID first) administration of any COVID-19/influenza vaccine in individuals >5 years were included. Two reviewers performed screening, and RoB assessment using RoB2.0. Non-inferiority (NI) margins were defined as GMR lower CI >0.67 [HAI titers]; >0.80 [Anti-Spike IgG]. GMRs were pooled using REML random-effects models (Stata v18.5) and RR (RevMan 5.4.1) for solicited local (pain, redness, swelling) & systemic AEs within 7 days (fever, fatigue, headache, myalgia, chills, nausea, arthralgia).

**Figure:**
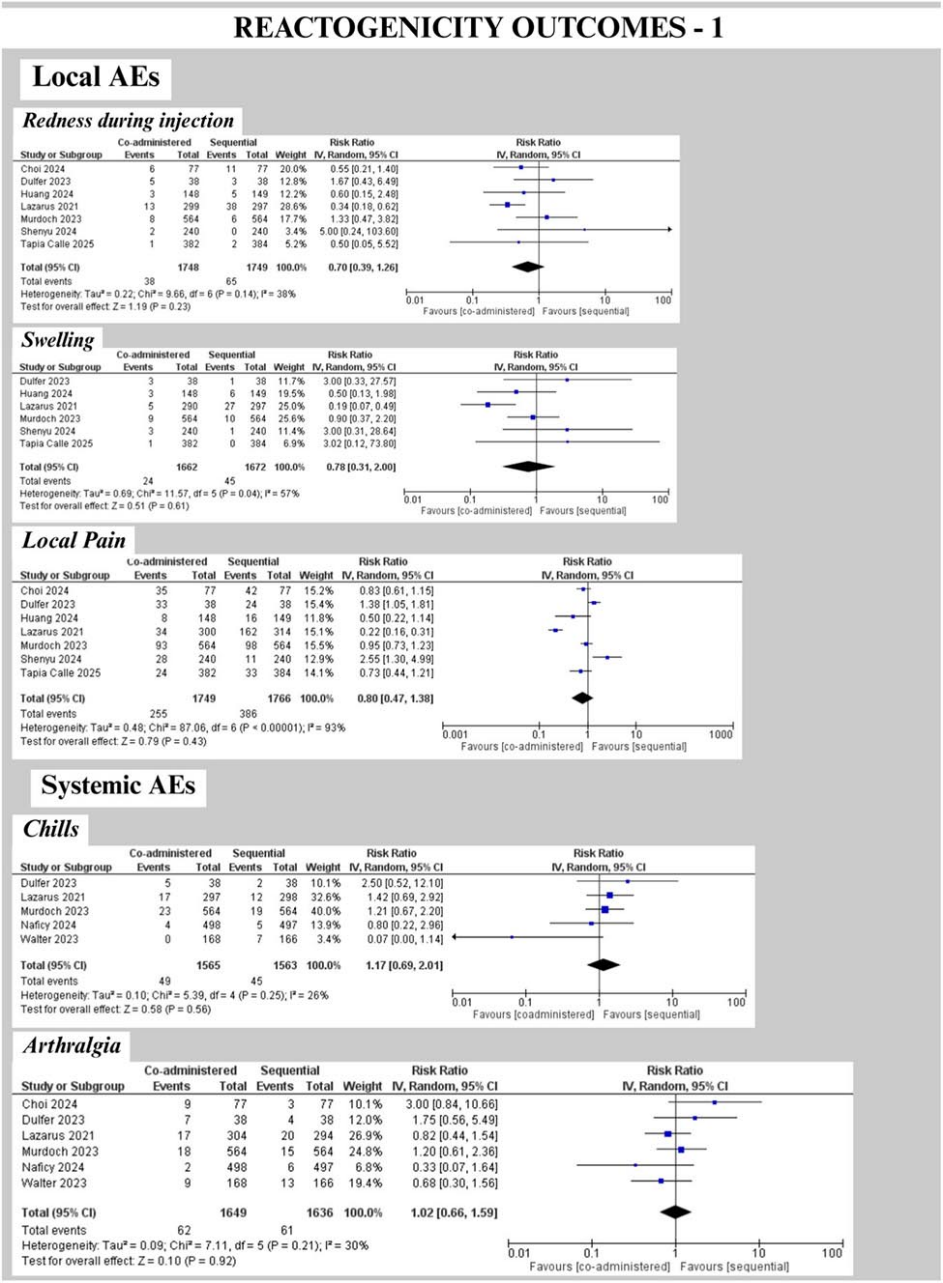

**Figure:**
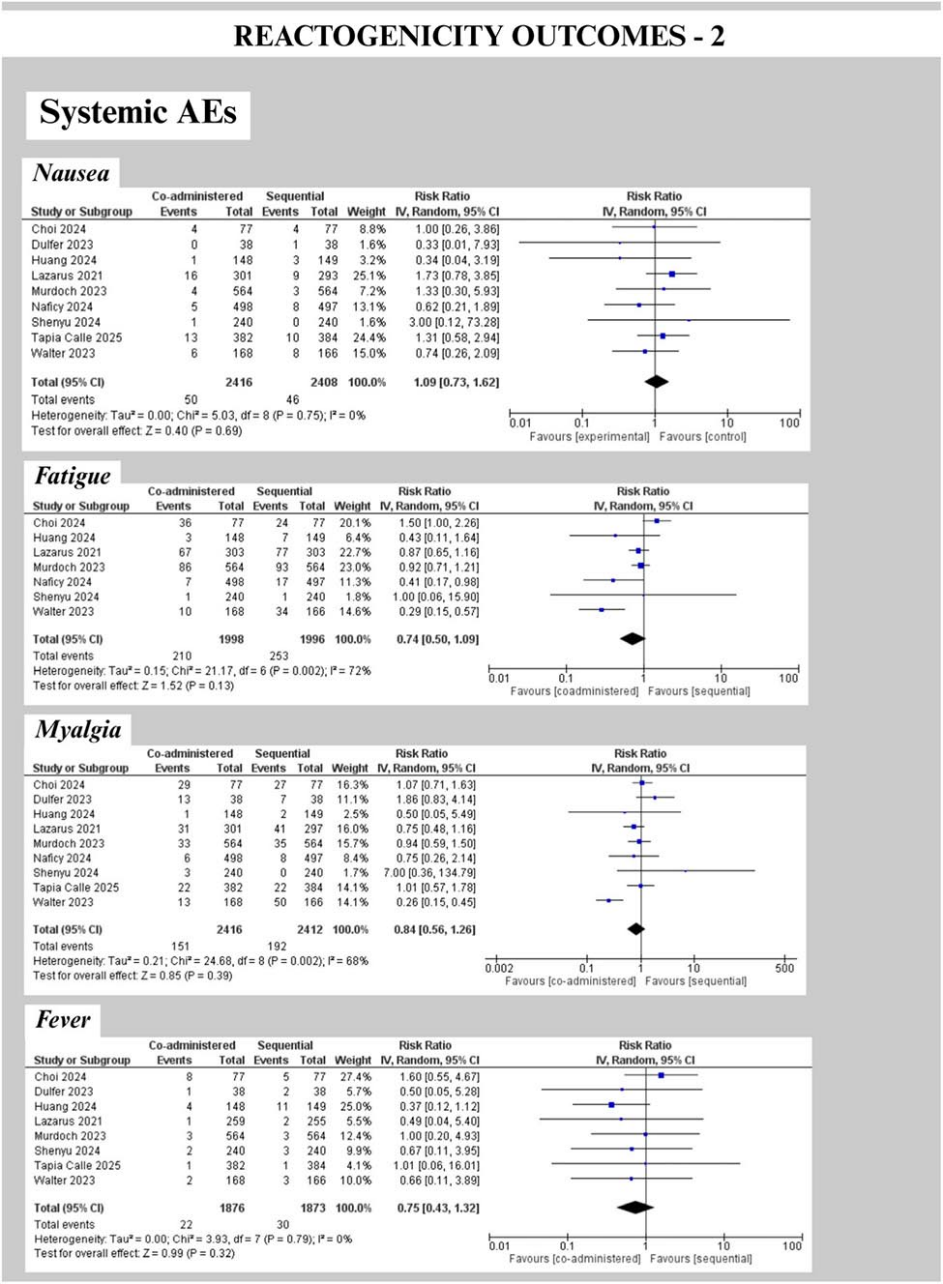

**Results:**

Nine RCTs (N= 4776) were included. There was comparable COVID-19 seropositivity but variable influenza serostatus at baseline. Co-administration modestly reduced anti-spike IgG GMRs versus sequential; 0.89 (0.78–1.02, p=0.09) for COVID-19-first and significantly 0.86 (0.81–0.92, p< 0.001) for influenza-first schedules, but lower CI met NI threshold. Influenza HAI titers’ pooled GMRs met NI criteria for all strains (A/H1N1 0.94 [0.88–1.00]; A/H3N2 0.96 [0.89–1.03]; B/Victoria 0.95 [0.90–1.00]; B/Yamagata 0.96 [0.91–1.01]). Solicited AEs showed no significant differences between groups (Fig. 4). Serious AEs were rare and comparable.

**Conclusion:**

Co-administration shows comparable safety and non-inferior immunogenicity versus sequential administration, despite modest reduction in Anti-Spike IgG Antibodies. It should be promoted, especially for flu vacinees.

**Disclosures:**

All Authors: No reported disclosures

